# Evaluating Outcomes Used in Cardiothoracic Surgery Interventional Research: A Systematic Review of Reviews to Develop a Core Outcome Set

**DOI:** 10.1371/journal.pone.0122204

**Published:** 2015-04-01

**Authors:** Carina Benstoem, Ajay Moza, Rüdiger Autschbach, Christian Stoppe, Andreas Goetzenich

**Affiliations:** 1 Department of Thoracic and Cardiovascular Surgery, University Hospital RWTH Aachen, Aachen, Germany; 2 Department of Anaesthesiology, University Hospital RWTH Aachen, Aachen, Germany; Harefield Hospital, UNITED KINGDOM

## Abstract

**Background:**

When planning clinical trials, it is a key element to choose appropriate outcomes that ensure the comparability of effects of interventions in ways that minimise bias. We hypothesise that outcome measures in cardiothoracic surgical trials are inconsistent and without standard. Therefore, comparing the relative effectiveness of interventions across studies is problematic. We surmise that cardiothoracic research has focused habitually on the identification of risk factors and on the reduction of adverse outcomes with less consideration of factors that contribute to well being and positive health outcomes (salutogenesis).

**Methods and Findings:**

We conducted a systematic review of reviews to determine both the type and number of outcomes reported in current cardiothoracic surgery interventional research, in order to identify a list of potential outcomes for a minimum core outcome set (COS). Special focus was placed on outcomes that emphasise salutogenesis. We interpreted salutogenic outcomes as those relating to optimum and/or positive health and well being. We searched Issue 7 (July 2014) of the Cochrane Database of Systematic Reviews. Systematic reviews of randomised trials on non-minimal-invasive off- or on-pump cardiothoracic surgery (elective and emergency, excluding transplants) investigating pre-, intra- or postsurgical interventions related to the outcome of the procedure were eligible for inclusion. We excluded protocols and withdrawn systematic reviews. Two review authors extracted outcome data independently. Unique lists of salutogenically and non-salutogenically focused outcomes were established. 15 systematic reviews involving 371 randomized trials and 58,253 patients were included in this review. Applied definitions of single and composite endpoints varied significantly, and patient-centred, salutogenically focused outcomes were seldom reported. One third of included reviews did not assess patient-centred outcomes at all; all other reviews were unable to perform meta-analyses due to an absence of data or heterogeneity in outcome measures. This compares to 36 non-salutogenically focused outcome domains representing 121 individual non-salutogenically focused outcomes, whereof 50% were assessed only once. Measures of mortality, cerebrovascular complications and hospitalisation were reported most frequently. Two reviews chose a composite endpoint as primary outcome. Pooled analysis of composite endpoints was not possible, as the required data was not reported per patient in all components.

**Conclusion:**

In cardiothoracic surgical trials, choice and definition of non-salutogenically focused single and composite outcomes are inconsistent. There is an absence of patient centred, salutogenically focused outcome parameters in cardiac trials. We recommend the development of a core outcome set of salutogenically focused and non-salutogenically focused outcomes for cardiothoracic surgical research.

## Introduction

When planning clinical trials, it is essential to choose appropriate outcomes to assure the comparability of effects of interventions in ways that minimise bias. However, there is a growing body of evidence indicating that inadequate attention has been paid to the outcomes measured in clinical trials; choice and definitions of outcome measures across trials vary considerably [1,2]. This problem is well recognised by systematic reviewers, as inconsistencies and heterogeneity in outcome reporting limits the ability of research synthesis [1,3–5].

Patients scheduled for cardiac surgery are at significant risk for the development of major adverse events during the postoperative course. Although surgical outcome has significantly improved over the last decade, cardiac surgery is generally associated with high incidences of myocardial, neurological and renal dysfunction, which account for up to 10% mortality, depending on the complexity of the surgical procedure, age, sex and co-morbidities [6–10]. Hence, coronary heart disease is one of the major contributors that cause the highest mortalities and burden of diseases worldwide [11]. Traditionally, trials in cardiac surgery traditionally focused on major outcome variables such as mortality, myocardial infarction or stroke, with little minimal consideration for what should be defined as optimum, for whom, and in what context. Although infrequent, serious adverse events are regularly measured, resulting in the need for large and cost intensive clinical trials to reliably detect true treatment effects of evaluated interventions. Only a small minority of outcomes, if any, appear to be patient-centred, promoting health and focusing on salutogenesis (lat. salus = health, gr. genesis = origin).

With the major focus being the reduction of severe adverse events, it is evident that there is little focus of what contributes to, or enhances, health and the well being of cardiac patients or how salutogenically focused outcomes could complement cardiothoracic surgical research. The term salutogenesis describes an approach focussing on factors that promote health and personal well being rather than on factors that cause diseases. It is a stress resource orientated concept, first introduced by Aaron Antonovsky in the late 1990’s [12], which focuses on resources, as well as maintaining and improving the movement towards health. It is the opposite of the pathogenic concept, where the focus is on obstacles and deficits.

For nearly thirty years, the World Health Organisation (WHO) [13] has advocated the promotion of health, rather than the avoidance of factors that cause illnesses. Accordingly, the WHO defines the promotion of health as “*the process of enabling people to increase control over*, *and to improve*, *their health*. *To reach a state of complete physical*, *mental and social well being*, *an individual or group must be able to identify and to realize aspirations*, *to satisfy needs*, *and to change or cope with the environment*. *Health is*, *therefore*, *seen as a resource for everyday life*, *not the objective of living*. *Health is a positive concept emphasizing social and personal resources*, *as well as physical capacities*. *Therefore*, *health promotion is not just the responsibility of the health sector*, *but goes beyond healthy life-styles to well being*“. However, we hypothesise that cardiothoracic research continues to focus on factors that cause diseases, how those factors could be influenced, and how the diseases can be treated.

Recent publications strongly recommend the development and use of agreed core outcome sets (COS), which should be measured and reported as a minimum in all trials for a specific clinical area [5,14], also compare http://www.comet-initiative.org. Minimum core outcome sets are well established in several clinical areas [16–18], which led to major breakthroughs in the treatment of cancer patients, and in rheumatology.

In the early 1970’s, the WHO became aware of this enduring problem. Two meetings were held (1977 in Turin and 1979 in Brussels) in order to develop a “common language” to describe cancer treatment, and to agree upon internationally acceptable general principles for evaluating data [16,17] that assured the comparability of results across trials. Essential details of patient characteristics or the therapy applied were guaranteed. It is apparent that cancer treatment still benefits from this early approach. The OMERACT (Outcome Measures in Rheumatology) is an independent initiative of international health professionals interested in outcome measures in rheumatology, who held their first conference in 1992 in Maastricht, with the aim to reach a consensus on core outcome criteria for clinical trials in rheumatoid arthritis [18], also compare http://www.omeract.org. The OMERACT Initiative is still very active today; focusing on different appearances of the disorder as well as patient reported outcomes (PRO’s) and quality of life assessment. The OMERACT Initiative is an impressive example of how patient stakeholders can influence research. After ten years, the OMERACT Initiative had to re-evaluate their core outcome set, despite of its success. The new core set omitted several outcomes of major importance to patients. One of these was fatigue, which is now included in the current version of the core outcome set on rheumatoid arthritis.

A minimum COS for cardiothoracic surgical research would address the problems depicted above. However, a recently published systematic review [15] on available core outcome sets for comparative effectiveness research has demonstrated that no COS exists for trials investigating pre-, intra- or postsurgical interventions in conventional cardiac surgery. As a consequence, we evaluated current clinical research on non-minimal-invasive off or on-pump cardiothoracic clinical trials (elective and emergency surgeries, excluding transplants) investigating pre-, intra- or postsurgical interventions to determine the type and number of outcomes reported by means of a systematic review of reviews. Furthermore, we assessed to what extent outcomes in cardiothoracic surgical clinical trials are patient-centred and reflect patients’ perception, interpretation or evaluation of their condition and quality of care and if endpoints focused on salutogenesis. The results of this review serve the purpose of providing the basis for developing a minimum core outcome set for cardiothoracic surgical research.

## Methods

In preparation of this systematic review, a protocol was composed (S1 Protocol). The review did not meet the inclusion criteria for PROSPERO, and therefore we were unable to register prospectively. However, a summary of the protocol is available at the COMET database (http://www.comet-initiative.org/studies/details/630?result=true). The conduct and reporting of this review adheres to, as much as practicable, the standards of the Cochrane Collaboration as outlined in the Cochrane Handbook [19] and the PRISMA checklist of reporting of systematic reviews [20], S1 Checklist.

### Study selection

#### Inclusion and exclusion criteria

We considered systematic reviews of randomised controlled trials for non-minimal-invasive off or on-pump cardiothoracic clinical trials (elective and emergency surgeries, excluding transplants) investigating any pre-, intra- or postsurgical interventions (participants > 18 years of age) for inclusion. Pre-, intra- and postsurgical interventions are defined as any intervention that occurred before, during or after cardiac surgery and were related to the outcome of the procedure. We excluded protocols for systematic reviews and systematic reviews that had been withdrawn.

#### Identification of relevant studies

**Fig 1:**
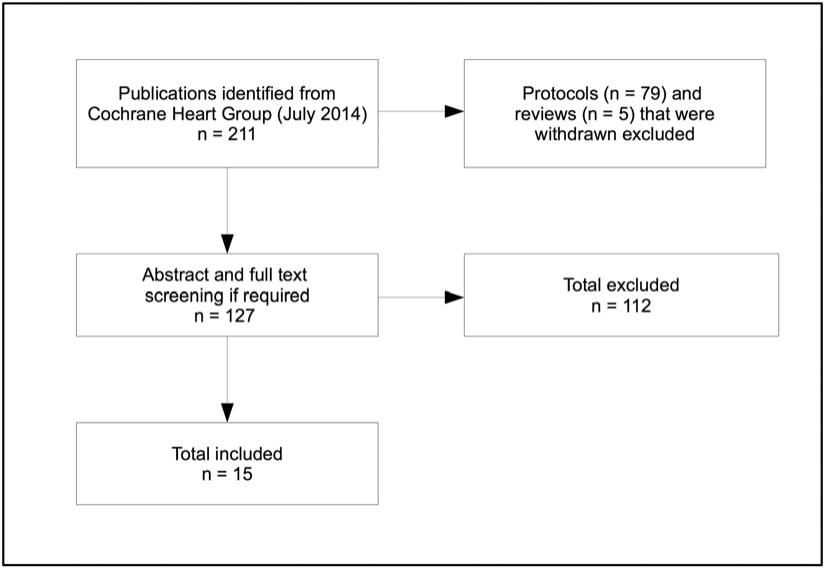
Screening and selection of reviews for inclusion.

We searched Issue 7 (July 2014) of the Cochrane Database of Systematic Reviews for all reviews published by the Cochrane Heart Group, using the advanced search option limited to “all text” and reviews published by the Cochrane Heart Group using “*” in the search box. Citations and abstracts were exported to EndNote X4 (Thomson Reuters, PA, USA). Each citation was then independently reviewed by two authors for inclusion via two complementary screening levels: level 1 = title or title and abstract screening, level 2 = full-text screening of citations judged relevant or considered relevant during screening level 1. Any disagreement of our judgements as to whether a review should be included was resolved through discussion and, if required, consultation with the team. Fig 1 provides a flow diagram detailing the process of selecting systematic reviews for inclusion and displaying the results.

### Data collection and management

**Fig 2:**
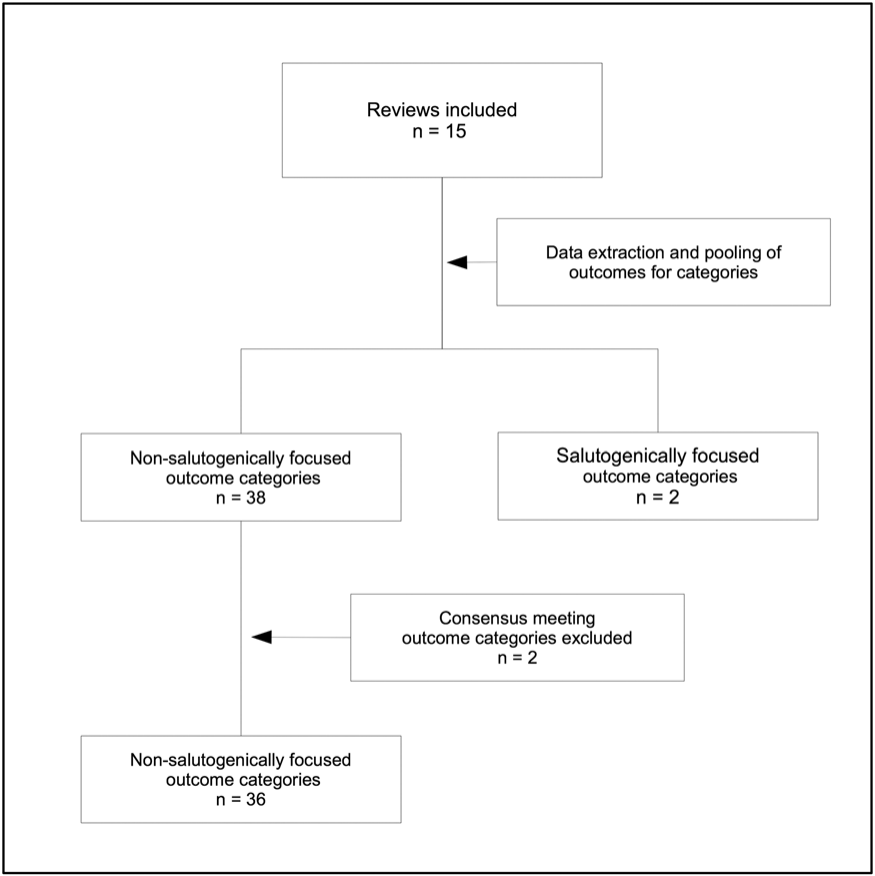
Data extraction process.

Data was independently extracted from each included review by two review authors using a purposively pre-developed data extraction form. Any disagreement was resolved through discussion and, if required, consultation with the team for consensus. For the purpose of this review, we adopted the definition of a salutogenically focused outcome introduced by Smith and colleagues [21], which was channelled by the characteristics of the “salutogenesis umbrella” [22]. A salutogenically focused outcome was thereby defined as a positively phrased outcome reflecting positive health and well being, rather than illness or adverse events prevention or avoidance. Within pair discussions, each individually extracted outcome was rated as either i) salutogenically focused or ii) non- salutogenically focused, in accordance to this definition. Any disagreement was resolved through discussion and, if required, consultation with the team for consensus. As a result, unique lists of salutogenically focused and non-salutogenically focused outcomes were established. In a subsequent step, outcome domains were determined accordingly during a consensus meeting with all team members. Fig 2 describes the data extraction process. If an outcome was reported more than once, we summarised obtained data to display the variety of definitions used per outcome domain.

## Results

We identified 15 systematic reviews that matched our inclusion criteria, presenting data on 371 randomised controlled trials. The trials were performed between 1971 and 2013, comprising statistics on 58,253 participants. One third of the included systematic reviews evaluated a pre-surgical intervention (n = 5), and each of the four publications presented data for an intra- or postsurgical intervention. Two reviews were not limited to a pre-, intra- or postsurgical timeframe. Following this process, we identified two salutogenically focused outcomes (Table 1), collapsing into two salutogenically focused outcome domains. For these two salutogenically focused outcomes (quality of life reported nine times, beneficial events- not specified by review authors and reported only once), none of the included systematic reviews were able to perform a meta-analysis due to lack of available data and inconsistency in outcome measurement. One third of included systematic reviews did not assess patient-centred or salutogenically focused outcomes at all.

**Table 1:** Unique lists of salutogenically focused outcome domains.

Salutogenically focused outcome domains	No. of times individual outcome was reported	No. of times individual outcome was analysed in meta-analysis
Quality of life	9	0
Beneficial events (not specified by review authors)	1	0

In contrast, we were able to extract data of 121 individual non-salutogenically focused outcomes reported in the included reviews. Those 121 unique non-salutogenically focused outcomes collapsed into 36 outcome domains. Overall, 50% of reported outcomes were reported only once. Table 2 displays these results, and states how many times the individual outcome was reported. Major outcome variables (mortality, cerebrovascular complications such as stroke and myocardial infarction) and outcomes relating to hospitalisations were reported most frequently. Table 3 shows the variety of outcome definitions used per domain for outcomes that were reported more than once. Utilised definitions and choice of outcomes were inconsistent for all outcome domains, e.g. for the non-salutogenically focused outcome domain *“Mortality”*, 13 different definitions and/or terms were used in 15 systematic reviews. This can be transferred to all other non-salutogenically focused outcome domains accordingly. Two of the included systematic reviews chose a composite outcome as a primary endpoint; both composite endpoints varied significantly. Both systematic reviews were unable to perform a pooled analysis since necessary data was not reported on patient level for all composites of the combined endpoint.

**Table 2:** Unique lists of non-salutogenically focused outcome domains.

Non-salutogenically focused outcome domain	No. of times individual outcome was reported
Mortality	18
Hospitalisation (length of hospital stay, length of ICU stay re-admission)	14
Cerebrovascular complications (stroke, infarction)	12
Economic outcomes / costs	7
Renal complications	7
Adverse events	5
Haemorrhagic complications (cardiac tamponade, bleeding, need for blood transfusion)	5
Measures of pulmonary function	5
Heart rhythm disturbances	5
Myocardial infarction	5
Chest tube (clearance, blockage, output volume, suspicious alternation)	4
Re- thoracotomy	3
Pulmonary complications or dysfunction	3
Composite outcomes	2
Infection	2
Inotropic use	2
Time to extubation	2
Intra aortic balloon pump use	2
Morbidity (not specified)	1
Incidence of modifiable coronary risk factor (smoking behaviour, blood lipid levels, blood pressure)	1
Neurological complications	1
Blood transfusion	1
Physical function measures	1
Coronary re-intervention	1
Complications of angiography or revascularization i.e. bleeding, procedure-related myocardial infarction or stroke	1
Low output syndrome	1
Limb ischemia	1
Haemodynamic parameters	1
Incidence indicators impending cardiac tamponade	1
Cardiovascular events	1
Pericardial effusion	1
Duration of follow up	1
Coronary re-stenosis	1
Measures of uptake / adherence to rehabilitation and lifestyle	1
Refractory angina	1
Thromboembolic events (other than cerebrovascular)	1

**Table 3:** Variety of outcome definitions used per outcome domain (if reported more than once).

Non-salutogenically focused outcome domain	Variety of outcome definitions used per domain in included studies
Mortality	Mortality
	Mortality rate
	Cardiovascular mortality rate
	Postoperative respiratory mortality
	Perioperative death not caused by stroke
	Mortality all causes
	Death all causes
	Total mortality
	Post- operative in-hospital all cause mortality (within three months)
	Post-operative all-cause mortality excluding inpatient mortality <30 days
	Mortality within the commonly accepted definitions of either prior to discharge or within 30 days
	All-cause mortality (mortality distribution and rates within the commonly accepted limits of either to discharge, within 30 days, 6 months and 1 year)
	Short-term post-operative mortality (i.e. in-hospital or 30-day mortality)
Hospitalisation (length of hospital stay, length of ICU stay re-admission)	Length of hospital stay
	Postoperative days in hospital
	Length of ICU stay
	Re- hospitalisation for acute coronary syndrome
Cerebrovascular complications (stroke, infarction)	Stroke
	Incidence of stroke or cerebrovascular accident
	Clinical evidence of cerebrovascular accident or stroke
	Neuroradiological evidence of brain infarction
	Neuropsychological testing for cognitive deficits
	Neurological deficits identified by neurological examination
	Trans-cranial doppler estimates of micro-emboli
	Biochemicalmarkers for cerebral damage (e.g. S-100 protein, neurospecific endolase)
	Subjective complaints of behaviour or memory change from patient or family
	Non-fatal cardiovascular events (re-infarction, re-occlusion and subsequent re-revascularization, stroke, recurrent ischemia), (hierarchical lower ranked endpoint
Economic outcomes / costs	Cost of treatment during hospital stay
	Health service utilisation
	Costs
	Economic costs
	Costs of care; cost-benefit, cost-effectiveness
Renal complications	Renal failure
	Renal insufficiency
	All forms of acute kidney injury (AKI), as defined by KDIGO 2012
	Validated renal injury scale, e.g. Acute Kidney Injury Network (AKIN) (Mehta 2007) or Risk, Injury, and Failure; and Loss, and End-stage kidney disease (RIFLE) criteria (Bellomo 2004)
	Use of continuous veno-venous haemo-filtration (CVVH)
	Hemofiltration requirements
Adverse events	Adverse events
	Adverse effects related to therapy
	Adverse events (serious and non-serious)
Haemorrhagic complications (cardiac tamponade, bleeding, need for blood transfusion)	Bleeding
	Gastro-intestinal bleeding
	Major haemorrhagic complications
	Incidence of cardiac tamponade—early (in first eight hours)
	Incidence of cardiac tamponade—late (after eight hours)
Measures of pulmonary function	Vital capacity (ml)
	Forced expiratory volume in one second (ml) (FEV1)
	Arterial Oxygenation (Partial pressure of arterial oxygen per inspired oxygen fraction (PaO2/FiO2)
	Respiratory muscle strength: MIP maximal expiratory pressure MEP
	Functional capacity: six minute walk test
Heart rhythm disturbances	Occurrence of atrial fibrillation
	Atrial fibrillation- any type
	Post-operative atrial fibrillation
	Incidence of atrial fibrillation or supraventricular tachycardia
Myocardial infarction	Myocardial infarction
	Perioperative non fatal myocardial infarction
	Myocardial infarction (fatal or non-fatal)
Chest tube (clearance, blockage, output volume, suspicious alternation)	Incidence of chest tube blockage
	Incidence of successful chest tube clearance
	Incidence of suspicious alteration in chest tube drainage pattern
	Absolute volume of chest tube output
Re- thoracotomy	Re- thoracotomy
	Incidence of re-opening the chest for bleeding
	Incidence of re-opening the chest for tamponade
Pulmonary complications or dysfunction	Atelectasis: radiographic, tomographic or bronchoscopic diagnosis and/or clinical signs with acute respiratory symptoms, for example dyspnoea, cough, abnormal lung sounds
	Occurrence of postoperative pulmonary complications grades 2, 3 or 4
Composite outcomes	Combined event rate or event free survival (e.g. major adverse cardiac events, major adverse cardiac and cerebrovascular events, target vessel failure or other composites of the events listed below); death (both cardiac and non-cardiac death); acute myocardial infarction (AMI); target vessel revascularisation (TVR); target lesion revascularisation (TLR); repeat treatment (PTCA, stent or CABG)
	Composite end-point, consisting of the following: all-cause mortality (in-hospital); fatal and non-fatal myocardial infarction (defined as: ECG changes, echocardiological changes, disproportionate elevation of troponines); pulmonary complications (including pulmonary edema and/or infection)
Infection	Infection
	Infectious complications
	Acute respiratory infection (pneumonia)
Inotropic use	Inotropic use
	Inotropic requirements
Time to extubation	Time to extubation
	Ventilatory requirements
Intra aortic balloon pump use	Intra-aortic balloon pump use (IABP) as markers for myocardial damage
	Intra-aortic balloon pump use (IABP)—related post-interventional complications

## Discussion

To our knowledge, this is the first approach to determine the type and number of salutogenically focused and non-salutogenically focused outcomes reported in current cardiothoracic surgery interventional research. In the 371 randomised controlled trials (n = 58,253) comprised in the 15 systematic reviews included in our study, we revealed that patient centred, salutogenically focused outcomes were seldom reported. Measures of quality of life (n = 9) and beneficial events (not specified by review authors, n = 1) were intended, but analysed in none of the meta-analyses due to lack of data in included studies, heterogeneity in outcome measurement and inconsistency in reporting. The near complete absence of salutogenically focused outcomes contrasts to a unique list of 36 non-salutogenically focused outcome domains representing 121 individually reported outcomes across all reviews. Major outcome variables (mortality, hospitalisations, cerebrovascular complications such as stroke and myocardial infarction) were analysed and published most commonly. Utilised definitions of single and composite endpoints showed an alarming inconsistency for the “same” outcome. Overall, 50% of all outcomes had appeared only once in included reviews.

The findings of our review support the hypothesis that the effectiveness of cardiothoracic surgery interventional research is measured against adverse effects, rather than increases in measures of health and well being. Patient-centred, salutogenically focused outcome parameters are not currently an inherent part of cardiothoracic clinical research. Avoidance of adverse events is a crucial element when exploring effectiveness of interventions for cardiothoracic surgical patients, and should remain an important component of cardiac surgical trials. However, the consistent, exclusive focus on risk reduction continues to form the basis for policy and practice development, without taking patients’ perception or evaluation of their condition, or quality of care and quality of life into consideration. When taking the contemporary curative nature of cardiothoracic surgery into account, it is vital that the perspective of all stakeholders—above all the patients’ perspective—is considered when deciding on outcome measurement. Here, the phrasing of clinical endpoints chosen by review authors to report on “any beneficial (…) events related to this review” [23] already expresses an awareness of the absence of commonly used patient-centred, positive health related outcomes. Simultaneously, the inability to encounter the problem becomes apparent.

At present, 3.127 clinical trials are registered at the “International Clinical Trials Registry Platform” (ICTRP) of the World Health Organisation [24], investigating cardiovascular diseases involving thousands of patients and costing millions of research funding. Without uniformity, internationally recognised definitions of clinical endpoints, and adherence to a minimum core outcome set for a certain clinical field, clinical trials will continue to produce avoidable waste in the production and reporting of research [4]. This impressive number of actively recruiting clinical trials in the field of cardiovascular disease highlights the urgent need for the minimum COS we propose.

Furthermore, we were able to demonstrate a considerable inconsistency in choice and definition of single and composite outcomes in cardiothoracic interventional surgical trials. Even for major outcome variables (mortality, myocardial infarction or stroke), which are measured most frequently in cardiac trials, no approved definition is internationally recognised. The terminology used, while presumably aiming to characterise the same variable, is deceptive and misleading (e.g. death all causes, mortality, total mortality or mortality all causes). This is further complicated by combination with a certain period of time (e.g. 28-day mortality, 30-day mortality, in-hospital mortality). Again, the wording of systematic reviewers to analyse “mortality within the commonly accepted definitions of either prior to discharge or within thirty days” [25] as a primary endpoint shows the major impact of the problem depicted for systematic reviewers and data syntheses, which aim to improve the treatment of cardiac surgical patients. Since standardised reports are crucial for meta-analyses and research synthesis, inconsistency and heterogeneity in outcome reporting continues to have significant influence far beyond the boundaries of a single clinical trial. It continues to impact treatment recommendations in guidelines, and affect clinical practice. Consequently, a standardised, salutogenically focused and non-salutogenically focused outcome evaluation is needed. This work highlights the need, and comprises the first element, to develop a minimum core (salutogenically focused and non- salutogenically focused) outcome set for cardiothoracic interventional surgical research. To date, the results of this systematic review cannot conclude direct implications for cardiothoracic surgery interventional research. However, it is our intention to reach consensus on endpoints to be included in the core outcome set via an eDelphi process in a subsequent step. The list of outcomes identified by this systematic review will provide the basis of clinically endpoints potentially included in the anticipated COS.

### Limitations of the present review

Focusing so exclusively limits our review to a select patient group and to measurements of effectiveness against outcomes specific for a certain period. However, our review was not intentionally focused on a specific population, nor on any particular intervention *per se*, but rather on reported single and composite endpoints and whether they were salutogenically focused or not. Although our search was limited to randomised controlled trials included in systematic reviews published by the Cochrane Heart Group (371 RCTs, n = 58,253), we were able to clearly demonstrate the necessity for the postulated minimum core outcome set, and we believe that all of our recommendations are justified on the basis of the evidence we have cited.

## Conclusion

There is an absence of salutogenically focused outcome parameters in cardiothoracic surgery interventional research. In the present review, we highlighted inconsistency and heterogeneity in reporting clinical endpoints of cardiac surgical studies. We recommend the development of a minimum core outcome set of salutogenically focused and non-salutogenically focused outcomes for intervention-based cardiothoracic surgical research.

## References of Included Studies

Arsenault KA, Yusuf AM, Crystal E, Healey JS, Morillo CA, Nair GM, Whitlock RP. Interventions for preventing postoperative atrial fibrillation in patients undergoing heart surgery. Cochrane Database of Systematic Reviews 2013, Issue 1. Art. No.: CD003611. DOI: 10.1002/14651858.CD003611.pub3.

Bakhai A, Hill RA, Dundar Y, Dickson RC, Walley T. Percutaneous transluminal coronary angioplasty with stents versus coronary artery bypass grafting for people with stable angina or acute coronary syndromes. Cochrane Database of Systematic Reviews 2005, Issue 1. Art. No.: CD004588. DOI: 10.1002/14651858.CD004588.pub3.

Karmali KN, Davies P, Taylor F, Beswick A, Martin N, Ebrahim S. Promoting patient uptake and adherence in cardiac rehabilitation.CochraneDatabase of SystematicReviews 2014, Issue 6.Art.No.:CD007131.DOI: 10.1002/14651858.CD007131.pub3.

Dieleman JM, van Paassen J, van Dijk D, Arbous MS, Kalkman CJ, Vandenbroucke JP, van der Heijden GJ, Dekkers OM. Prophylactic corticosteroids for cardiopulmonary bypass in adults. Cochrane Database of Systematic Reviews 2011, Issue 5. Art. No.: CD005566. DOI: 10.1002/14651858.CD005566.pub3.

Freitas ERFS, Soares BGO, Cardoso JR, Atallah ÁN. Incentive spirometry for preventing pulmonary complications after coronary artery bypass graft. Cochrane Database of Systematic Reviews 2012, Issue 9. Art. No.: CD004466. DOI: 10.1002/14651858.CD004466.pub3.

Hoenig MR, Aroney CN, Scott IA. Early invasive versus conservative strategies for unstable angina and non-ST elevation myocardial infarction in the stent era. Cochrane Database of Systematic Reviews 2010, Issue 3. Art. No.: CD004815. DOI: 10.1002/14651858.CD004815.pub3.

Hulzebos EHJ, Smit Y,Helders PPJM, vanMeeterenNLU. Preoperative physical therapy for elective cardiac surgery patients. Cochrane Database of Systematic Reviews 2012, Issue 11. Art. No.: CD010118. DOI: 10.1002/14651858.CD010118.pub2.

Liakopoulos OJ, Kuhn EW, Slottosch I, Wassmer G,Wahlers T. Preoperative statin therapy for patients undergoing cardiac surgery. Cochrane Database of Systematic Reviews 2012, Issue 4. Art. No.: CD008493. DOI: 10.1002/14651858.CD008493.pub2.

Massel DR, Little SH. Antiplatelet and anticoagulation for patients with prosthetic heart valves. Cochrane Database of Systematic Reviews 2013, Issue 7. Art. No.: CD003464. DOI: 10.1002/14651858.CD003464.pub2.

Møller CH, Penninga L, Wetterslev J, Steinbrüchel DA, Gluud C. Off-pump versus on-pump coronary artery bypass grafting for ischaemic heart disease. Cochrane Database of Systematic Reviews 2012, Issue 3. Art. No.: CD007224. DOI: 10.1002/14651858.CD007224.pub2.

Rees K, Beranek-Stanley M, Burke M, Ebrahim S. Hypothermia to reduce neurological damage following coronary artery bypass surgery. Cochrane Database of Systematic Reviews 2001, Issue 1. Art. No.: CD002138. DOI: 10.1002/14651858.CD002138.

Spencer S, Tang A, Khoshbin E. Leukodepletion for patients undergoing heart valve surgery. Cochrane Database of Systematic Reviews 2013, Issue 7. Art. No.: CD009507. DOI: 10.1002/14651858.CD009507.pub2.

Theologou T, Bashir M, Rengarajan A, Khan O, Spyt T, Richens D, Field M. Preoperative intra aortic balloon pumps in patients undergoing coronary artery bypass grafting. Cochrane Database of Systematic Reviews 2011, Issue 1. Art. No.: CD004472. DOI: 10.1002/14651858.CD004472.pub3.

Unverzagt S, MachemerMT, Solms A, Thiele H, Burkhoff D, SeyfarthM, deWaha A, Ohman EM, Buerke M, Haerting J, Werdan K, Prondzinsky R. Intra-aortic balloon pump counterpulsation (IABP) for myocardial infarction complicated by cardiogenic shock. Cochrane Database of Systematic Reviews 2011, Issue 7. Art. No.: CD007398. DOI: 10.1002/14651858.CD007398.pub2.

Wallen MA, Morrison AL, Gillies D, O’Riordan E, Bridge C, Stoddart F. Mediastinal chest drain clearance for cardiac surgery. Cochrane Database of Systematic Reviews 2002, Issue 2. Art. No.: CD003042. DOI: 10.1002/14651858.CD003042.pub2.

## Supporting Information

S1 Protocol (PDF)

S1 Checklist (PDF)
