# Supplementary material for: Evaluating Outcomes Used in Cardiothoracic Surgery Interventional Research: A Systematic Review of Reviews to Develop a Core Outcome Set
Source: PLoS One. 2015 Apr 1;10(4):e0122204. doi: 10.1371/journal.pone.0122204 (PMC4382223; doi:10.1371/journal.pone.0122204)
Supplement: S1 Protocol — (PDF) [file pone.0122204.s001.pdf]

Work plan

Development of a core outcome set for clinical trials in adult heart surgery: procedures on heart valves or coronary arteries

Background

The World Health Organisation (WHO) defines a clinical trial as "any research study that prospectively assigns human participants or groups of humans to one or more health-related interventions to evaluate the effects on health outcomes" (WHO 2013). In dependence, it is a key element to choose appropriate outcomes when planning clinical trials to assure the comparability of effects of interventions in ways that minimise bias.

However, there is a growing body of evidence indicating that inadequate attention has been paid to the outcomes measured in clinical trials. The choice and definitions of outcome measures used by researchers varies considerably. This problem is well known to systematic reviewers as inconsistencies and heterogeneity in outcome reporting limit the ability of research synthesis. As a consequence, a significant number of identified matching studies are regularly excluded from meta-analyses reducing power and limiting the value of available evidence (Kirkham 2009). Furthermore, empirical research strongly determines that outcome-reporting bias (defined by the Cochrane Collaboration as "selective reporting of some outcomes but not others, depending on the nature and direction of the results", Cochrane Handbook, Chapter 10, Table 10.1.a) has significant impact on how the results of clinical trials are reported (Dwan 2008), which emphasises the need for appropriate (core) outcomes in a particular clinical field.

Recent publications (Clarke 2007, Williamson 2012, compare also comet-initiative.org) strongly recommend the development and the use of agreed core outcome sets (COS), which should be measured and reported as a minimum in all trials for a specific clinical area. An initial review of the literature showed that no such COS exists for interventional clinical trials regarding adult cardio thoracic surgery. Additionally, the majority of assessed outcomes appear to be exclusively focused on reducing adverse effects and identifying risk factors. Only the minority, if at all existing, seems to be patient centred and focused on salutogenesis.

Objectives of this thesis

To systematically review outcomes used in previous systematic reviews of randomised trials (adult heart surgeries, non-minimal-invasive off- or on-pump (elective and emergency, excluding transplants) investigating pre-, intra- or postsurgical interventions related to the outcome of the procedure) to identify a list of potential outcomes for a COS.

To identify patient-centred and salutogenic focused outcomes reported in included systematic reviews.

To develop a core outcome set for clinical trials on non-minimal-invasive off- or on-pump cardiothoracic surgery (elective and emergency, excluding transplants) investigating pre-, intra- or postsurgical interventions.

Methods

Identification of existing knowledge

This method for reviewing already existing knowledge is developed in consideration of quality standards provided by the Cochrane Collaboration (Higgins 2011) and recommendations for developing a COS (Williamson 2012). The following inclusion criteria need to be meet by studies to be considered as a basis for the potential list of outcomes for the COS:

Types of studies

Systematic reviews of randomised trials are considered for inclusion.

Types of participants

Adult patients (≥18 years of age) are included.

Types of interventions

The German modification of the “International Classification of Procedures in Medicine” (ICPM), the “Operationen- und Prozedurenschlüssel (OPS 5.35 - 5.36)“, provided the basis for operational procedures to be included in this systematic review (**non-minimal-invasive off- or on-pump cardiothoracic surgery (elective and emergency, excluding transplants**), which includes:

Any operational procedure on heart valves (aortic valve, mitral valve, pulmonary valve and tricuspid valve; also minimal invasive)

- Valvulotomy
- Valve replacement
- Replacement of artificial valve prostheses
- Valvuloplastics

Any operational procedure on coronary arteries

- Desobliteration of coronary arteries
- Any type of coronary artery bypass grafting (CABG)
- Any other revascularisation technique

Search methods

Search of the Cochrane Database of Systematic Reviews for all reviews published by the Cochrane Heart Group for reviews that fulfil the inclusion criteria.

Data extraction

The selection of studies will be in all cases performed blindly by two reviewers independently via two complementary screening levels: level 1 = title respectively title and abstract screening, level 2 = full-text screening. The reference manager software EndNote X4 will be used to access the search results. Each potentially eligible review identified as a result of the search strategy will be assessed by a colleague and the principal investigator. Any disagreement in their judgements as to whether a review should be included will be resolved through discussion or, if required, by consultation with a third person. Full text papers will be obtained for all potentially eligible studies for screening level 2. If one potential matching review is published in a language other than German or English, native speakers or fluent translators will be involved in the translation of studies. A data extraction sheet in Excel format will be developed to obtain relevant data. Data extraction will be performed by two review authors independently using the agreed from separately.

Identification of patient-centred and salutogenic focused outcomes

Aaron Antonovsky first introduced the concept of salutogenesis while studying the psychological impact of surviving concentration camps (Antonovsky 1987). Antonovsky explored how people who had experienced extremely stressful life events remained resilient and positive about their lives, which formed a new theoretical framework for health coined ‘salutogenesis’. A key component of salutogenesis is that of a “sense of coherence”, which postulates that an individual who can view the world as manageable (i.e. easily find resources for coping), comprehensible (perceived clarity, order and structure) and meaningful (has purpose) is more likely to view their life as coherent (Antonovsky 1987). In this sense Antonovsky proposed, that no matter how extreme an individual’s experience might be, they will have the ability to cope positively with adverse events. Salutogenesis was the first theory of its kind to explore health systematically in terms of movement along the health continuum, thereby eliminating a distinct dichotomy of being in a state of health or being in a state of disease.

Research regarding adult cardio thoracic surgery has focused traditionally on the reduction of adverse outcomes with little consideration for what is optimum, for whom and in what context; the prevention of adversity has habitually been the focus rather than the promotion of health. Such an interventionist approach suggests that there is little understanding of what contributes to or even enhances the health and the wellbeing of cardiothoracic adult patients and how salutogenically focused outcomes could substitute to adult cardio thoracic research. As a first step, current research needs to be evaluated to determine the type and number of salutogenically-focused reported outcomes regarding any operational procedure on the adult heart including heart valves (aortic valve, mitral valve, pulmonary valve and tricuspid valve; also minimal invasive) or the coronary arteries.

For the purposes of this thesis, a salutogenically-focused outcome is defined as an outcome reflecting positive health and wellbeing rather than illness or adverse event prevention or avoidance. The judgement whether or not a assessed outcome is salutogenically-focused or not will in all cases performed by two review authors independently, any disagreement in their judgements will be resolved through discussion or, if required, by consultation with a third person.

Development of the COS

The Delphi method is iterative and uses a series of rounds of data collection and analysis to condense the opinions of individuals into group consensus. Typically, it involves the use of sequential rounds of postal questionnaires that are designed to elicit participants' opinions on a particular topic. Responses to each round are collated, analysed, and redistributed to participants for further comment in successive rounds. It is chosen to conduct a eDelphi survey online, so as to facilitate international participation without the time lag between successive rounds associated with traditional postal surveys, to enable a relatively low cost structure, to increase data collection efficiencies, and to provide the potential for a higher response rate through rapid communication with participants. The survey will be conducted using the online survey software QuestionPro (http://www.questionpro.com).

For this COS it is intended to provoke opinions and to reach consensus about important outcomes to be included in cardiothoracic surgery on the adult heart including heart valves (aortic valve, mitral valve, pulmonary valve and tricuspid valve; also minimal invasive) or the coronary arteries.

Participants

Participation is sought from people within the following broad groups: adult patients in need or after cardiothoracic surgery with regard to any operational procedure on the adult heart including heart valves or the coronary arteries (= stakeholder involvement), cardio-thoracic surgeons, anaesthesiologists, nursing staff involved with adult cardiothoracic patients and researchers with expertise in this particular field of medical research.

An e-mail inviting participation will be send to the following groups, which was identified as relevant to the broad area of expertise regarding the subject under investigation: Deutsche Gesellschaft für Thorax-, Herz- und Gefäßchirurgie (DGTHG), Centres for Cardiothoracic Surgery in Germany, European Association for Cardio Thoracic Surgery (EACTS), American Association for Thoracic Surgery (AATS), Society of Thoracic Surgeons (STS), the Cardiothoracic Surgery Network (CTSNet), the Cochrane Heart Group and the Comet Initiative to approach researchers with knowledge in developing COS. Participants are then invited to use snowball sampling by forwarding the invitation to colleagues whom they regard as having the required expertise to substantially contribute to this eDelphi survey and the development of the COS. Those who additionally want to participate will be asked to respond with their name and e-mail address.

Pilot study

The initial instrument containing the outcomes identified by the above-mentioned systematic review of randomised controlled trials and systematic reviews of randomised controlled trials on adult patients undergoing any surgical procedure on heart valves (aortic valve, mitral valve, pulmonary valve and tricuspid valve; also minimal invasive) or the coronary arteries will be tested for clarity with a sample of 10 participants.

Consensus methods

It is intended to reach consensus on the COS of important outcomes to be included in cardiothoracic surgery on the adult heart including heart valves or the coronary arteries in a 3-round eDelphi exercise. The first round will contain all outcomes identified by the aforesaid systematic review; for better overview those outcomes will be presented in groups, e.g. general outcomes applicable to all patients, outcomes regarding only surgical procedures on the heart valves or the coronary arteries and exclusively patient-centred and salutogenically-focused outcomes.

Participants will be asked to rate the importance of each outcome listed using a 5-point Likert-type scale rating their importance for inclusion in a minimum set as: 1 = of no importance, 2 = of some importance, 3 = of moderate importance, 4 = very important, and 5 = extremely important. Participants

will also be asked to identify up to 2 ‘‘new’’ outcomes under each of the broad headings, which they judged to be relevant or important. The round 1 instrument will also included a short online demographic questionnaire and will invite participants to rate their perceived level of expertise in evaluating models of maternity care on a 7-point Likert-type scale (1 = low level of perceived expertise, 7 = high level of perceived expertise). A copy of each participant's response to every round will be e-mailed to them within 48 hours of completion of that round online. Each round had a response closing date 14 days after the date of invitation. An e-mail reminder will be send to anyone who had not responded by day 7.

In round 2, participants who responded to round 1 will be presented with outcomes retained after analysis of responses from round 1, where (a) the overall mean score for inclusion for that outcome will be greater than the mean score for all the outcomes combined and (b) the mean score for inclusion for that outcome will be greater than the mean score for all the outcomes combined for those participants who had rated their perceived level of expertise in evaluating cardiothoracic surgery as high (i.e., 6 or 7 on the Likert scale). Additional outcomes identified by participants in round 1 will be included if suggested by at least 2 participants. For each outcome retained from round 1, the overall group's mean rating and standard deviation will be presented. Participants will be asked to re-rate the importance of each outcome with knowledge of their individual and the group's previous ratings. In addition, participants will be asked to rate the newly identified outcomes from round 1. All ratings used the same Likert-type scale that was used in round 1.

In round 3, participants who responded to round 2 will be presented with outcomes retained after analysis of responses from round 2, where (a) the overall mean score for inclusion for that outcome will be greater than the mean score for all the outcomes combined and (b) 70 % or more of study participants will have rated their importance for inclusion as a ‘‘4’’ or ‘‘5’’ on the 5-point Likert-type scale used in round 2. Each of the outcomes in the round 3 instrument will again be presented together with the mean rating and standard deviation for the whole group, and participants will be asked to re-rate the importance of each item for inclusion in a minimum data set using the same Likert-type scale used in round 2. Outcome measures will be retained from round 3 if the group mean for that outcome will be greater than the mean score for all the outcomes combined and where 70 % or more of the participants gave an importance rating of 4 or 5 on the 5-point Likert-type scale.

Data Analysis

Data analysis will be performed using SPSS (Version x.xx). Mean and standard deviation will be rounded to 2 decimal places.

Implementation of core outcome set

It is intended to register this study with the COMET Initiative after approval of Dr. Goetzenich. The results of the systematic review and the COS will be published in two consecutive publications at English publishing, peer-reviewed journals.

References

Antonovsky A. (1987) “Unravelling the mystery of health: how people manage stress and stay well” Jossy-Bass, California.

Clarke M (2007) “Standardising outcomes for clinical trials and systematic reviews“ *BioMed Central*, Vol. 8, no. 39, pp.1-3

Dwan K, Altman DG, Arnaiz JA, Bloom J, Chan AW, Cronin E, Decullier E, Easterbrook PJ, von Elm E, Gamble C, Ghersi D, Ioannidis JP, Simes J, Williamson PR (2008) “Systematic review of the empirical evidence of study publication bias and outcome reporting bias” *PLoS One*, Vol. 3, Issue 8, pp. 1-31

Higgins JPT, Green S (editors). *Cochrane Handbook for Systematic Reviews of Interventions* Version 5.1.0 [updated March 2011]. The Cochrane Collaboration, 2011. Available from www.cochrane-handbook.org.

Kirkham JJ, Dwan KM, Altman DG, Gamble C, Dodd S, Smyth R, Williamson PR (2010) “The impact of outcome reporting bias in randomised controlled trials on a cohort of systematic reviews” *BMJ*, 340:c365, pp. 1-10

WHO (2013) “International Clinical Trials Platform (ICTRP)” (online). Available from: http://www.who.int/ictrp/en/ [Accessed 7. April 13]

Williamson PR, Altman DG, Blazeby JM, Clarke M, Devane D, Gargon E, Tugwell P (2012)
"Developing core outcome sets for clinical trials: issues to consider" *Trials*, Vol. 13, no. 132, pp. 1-8
